# Barriers and Promoters of Healthy Eating from the Perspective of Food Environment Perception: From Epidemiology to the Talking Map

**DOI:** 10.3390/ijerph22071109

**Published:** 2025-07-15

**Authors:** Bruna Aparecida Avelar, Anabele Pires Santos, Renata Adrielle Lima Vieira, Raquel De Deus Mendonça, Mariana Carvalho de Menezes

**Affiliations:** 1 School of Nutrition, Federal University of Ouro Preto, Ouro Preto 35400-000, MG, Brazil; bruna.avelar@aluno.ufop.edu.br (B.A.A.); anabele.pires@unifesp.br (A.P.S.); raquel.mendonca@ufop.edu.br (R.D.D.M.); 2 Health Sciences Center, Department of Nutrition, Federal University of Paraíba, João Pessoa 58051-900, PB, Brazil; renataadrielle.ufpb@gmail.com; 3 Department of Nutrition, Nursing School, Federal University of Minas Gerais, Belo Horizonte 30130-100, MG, Brazil

**Keywords:** perception, food environment, access to healthy foods, environmental barriers, focus groups, qualitative evaluation, food system

## Abstract

Background: Food environments can determine food choices, acting as barriers to or promoters of healthy eating. It is necessary to investigate individuals’ perceptions of those barriers and promoters of healthy eating in the food environment. Methods: This is a qualitative and quantitative study involving patients diagnosed with arterial hypertension. In the quantitative approach, a validated questionnaire for the Brazilian population, the Perceived Nutrition Environment Measures Survey, was used. For the qualitative approach, a talking map was applied in a focus group with guiding questions. Quantitative data were analyzed through simple relative frequency, and qualitative data through reports; subsequently, both were grouped into perceived barriers and facilitators. Results: Participants found high access to ultra-processed foods, strongly influenced by advertising in commercial establishments, as a barrier, as well as barriers related to changes in commensality habits and transformations in food systems. As promoting factors, access to fruits and vegetables was highlighted as favoring healthier food choices. The qualitative findings emphasized the importance of home gardens and foods sourced from family farming. Conclusions: This study found that individuals perceive high access to ultra-processed foods in their food environment, both in financial terms and availability, while reporting low access to fresh foods.

## 1. Introduction

The food environment can be understood as the physical, social, political, cultural, and economic context in which people interact [1]. It is a space of mediation between production chains and food choices, in which factors directly influence individuals’ diets, such as supply, accessibility, price, and food promotion [2].

Several models to classify and understand food environments have been proposed, among which we highlight those that make a distinction between the objective environment—which can be measured by physical and geographic indicators—and the perceived environment, which considers individuals’ subjective interpretations of the spaces in which they circulate and eat [3]. Although most public health assessments focus on objective measures [4,5], studies indicate that eating behaviors are guided by individual perceptions to a large extent, not only by the actual conditions of the environment [6,7,8]. In other words, food choices are shaped by the way individuals perceive the food environment around them [3].

In this sense, investigating the perceived food environment becomes a relevant strategy to understand factors that hinder or promote healthy eating practices in territories. Frequently identified barriers include easy access to ultra-processed foods (UPF), their intense advertising, and strategic placement at points of sale, factors associated with the increase in Chronic Non-Communicable Diseases (NCDs) [9,10,11,12,13]. On the other hand, the availability of natural foods, easy access to healthy options, and better socioeconomic conditions are recognized as promoters of adequate nutrition [14,15].

In addition to impacts on health, food is deeply interconnected with the food system because promoting healthy food environments also implies considering practices that reduce negative impacts on the environment, such as the emission of polluting gases and waste throughout the food chain [2,16]. Thus, individual perceptions of the food environment not only affect human health but also reflect and influence behaviors with environmental implications [3].

In view of this, understanding how individuals perceive the barriers and promoters of the food environment is essential to support territorial public policies. In the context of Primary Health Care (PHC), which works directly in the territories and with communities, this can strengthen inter-sectoral actions to promote adequate and sustainable nutrition. However, there are still few studies that explore the perceived food environment based on the articulation between qualitative and quantitative methods to explore the complexity of perceptions about the food environment.

Therefore, this study aims to understand the barriers and promoters of healthy eating perceived by PHC users in their food environment through subjective assessments in order to generate scientific evidence to support public policies that promote healthier and more sustainable food environments.

## 2. Materials and Methods

This is an exploratory and descriptive research study, with a qualitative and quantitative approach. It comes from the first phase of a larger nutritional intervention project with Primary Health Care (PHC) users with arterial hypertension (AH) in the municipality of Ouro Preto, Minas Gerais. The municipality belongs to the Iron Quadrangle region in Minas Gerais, one of the largest iron ore-producing areas in Brazil, with a population of 74,824 people; a demographic density of 60.06 inhabitants/km^2^, according to the 2022 demographic census; and a Municipal Human Development Index of 0.741, according to the 2010 demographic census [17,18].

The project was developed with PHC users and aimed to evaluate the effectiveness of a new methodology based on motivational interviewing and the guidelines of the Food Guide for the Brazilian Population, which is innovative in comparison with traditional methods with individual nutritional intervention.

### 2.1. Study Design and Participants

In order to quantitatively assess perceptions about the food environment, hypertensive healthcare system users monitored in six Family Health Strategies (FHS) in Ouro Preto, MG, were invited to participate in the project. The representative sample included individuals of both sexes, aged > 20 years, who had been diagnosed with hypertension and had answered the food environment questionnaires within 15 days after their first consultation with nutritionists. Additional details about the project and participant recruitment can be obtained from Avelar et al. (2023) [19].

Regarding the qualitative approach, this is a participatory research conducted by the application of the talking map technique through focus groups in the situational diagnosis stage prior to the HipertenSUS interventions. The participants were digitally contacted and invited by the community health agents of the 11 FHSs in the municipality. Confirmations were recorded in an attendance list, with a maximum of 13 participants per FHS, since it was a pandemic period, and health safety measures had to be adopted. In the end, 4 workshops were held in 4 FHSs.

### 2.2. Assessment of the Perceived Food Environment

In the quantitative approach, the questionnaire was applied between March and July 2022 to baseline participants, with an interval of 2 to 10 days after the first nutritional consultation, in order to minimize its possible interference with eating behaviors. The collection was conducted by previously trained nutritionists and students, with field supervision and using the KoBoCollect^®^ application on tablet computers version v1.30.1. Sociodemographic, economic, and food consumption data were obtained during the first consultation, and the questionnaire was applied on weekdays at different Basic Health Units (BHU) in the municipality.

**Figure 1:**
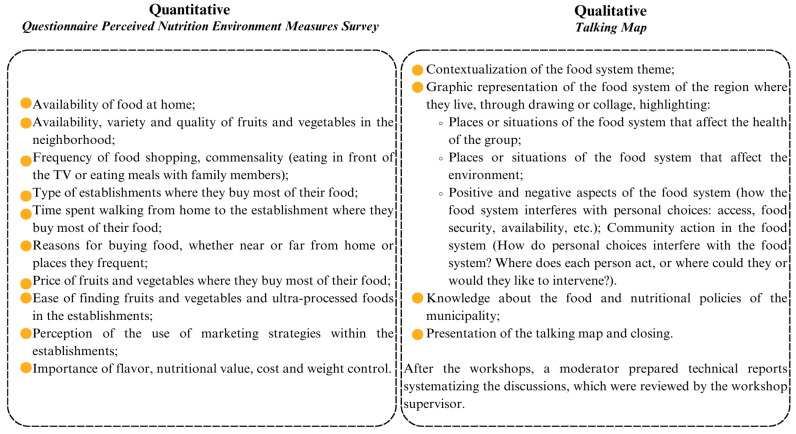
Selected questions from the Perceived Nutrition Environment Measures Survey for quantitative data and focus group dynamics for creating the talking map.

In order to assess perceptions about the food environment, the questions in Figure 1 were selected from the Perceived Nutrition Environment Measures Survey (NEMS-P), which is widely used in the international literature and translated and validated for Brazil [19,20,21], as shown in Figure 1. The selection of the questions was carried out with the objective of assessing the food environment from different domains, such as access, availability, and promotion of foods, present in the NEMS-P [20]. Hence, questions that addressed similar aspects were excluded in order to ensure greater objectivity and to avoid redundancies, prioritizing those that best capture the diversity and complexity of individual perceptions on the food environment. And later, the response categories were grouped in order to reduce them and facilitate the identification of responses that indicated whether something was considered important or not important. This step allowed for the assessment of individual perceptions regarding the availability and access to healthy foods in households and neighborhoods based on a classification theoretically grounded in two core constructs of the transtheoretical model of behavioral change [22]. The selected items reflect aspects directly related to self-efficacy (e.g., confidence in finding healthy foods in the neighborhood) and decisional balance (e.g., perceptions of prices, variety, and quality of available foods) [22].

In order to classify the foods mentioned in the NEMS-P, they were classified according to the NOVA system afterwards [23]. Thus, fruits and vegetables were classified as natural foods, while sweets, snacks, sugary drinks, and other items considered unhealthy were grouped together and called ultra-processed foods (UPF).

### 2.3. Assessment of the Perceived Food System Based on Talking Map Workshops

Data collection for the talking map was carried out through workshops held between November and December 2021 at BHUs. The talking map can be conceptualized as the perception of the individual or collective about the space in which they live; that is, the geographic space based on their experience, such as the presence of food establishments, squares, and churches [24]. This technique favors the emergence of aspects of daily food that would not be captured by structured instruments, since the individuals participate in the process from a dialogic, active perspective [25].

The following themes were used in guiding questions: food system; health; environment; food access, availability, and security; and public policies for food and nutrition.

The workshops were divided into five stages: (1) introduction of the participants; (2) presentation of the topic by showing the video “What is Healthy Eating?”, available on the YouTube channel of the Alliance for Adequate and Healthy Eating (https://www.youtube.com/watch?v=PFxwtzf8XW0, accessed on 10 November 2021); (3) development of the talking map; (4) presentation of the talking maps; and (5) final considerations.

During the workshops, dialogue and reflections among the participants on the eating practices experienced were encouraged to emerge, as were aspects of the users’ daily eating habits.

The script proposed by Matuk and Toledo (2018) was taken into consideration [24] to create the map. It included a question regarding knowledge about the city’s food and nutrition policies, as shown in Figure 1.

After the workshops, a moderator prepared technical reports systematizing the discussions, which were then reviewed by the workshop supervisor.

### 2.4. Data Analysis

Descriptive analyses of the sample were performed using simple relative frequency with the SPSS Statistics software (version 20.0).

The qualitative results of the talking map were organized into a report for subsequent grouping and documentary analysis. The reports were analyzed with an interdisciplinary, dialogic perspective [26].

The results obtained from both collections were analyzed and distributed between two major categories: barriers and promoters of healthy eating from the perspective of the PHC users’ perception.

This study was conducted in accordance with the guidelines established in the Declaration of Helsinki, and all procedures involving participants in research studies were approved by the Human Research Ethics Committee under protocol no. 5327958 of the Federal University of Ouro Preto, with Certificate of Presentation of Ethical Appraisal no. 42858120.9.0000.5150. The Free and Informed Consent Form (FICF) was signed in the first face-to-face interview.

## 3. Results

### 3.1. Quantitative

In this approach, the final sample consisted of 176 people who met the inclusion criteria. Most participants were female (79.0%); aged between 40 and 59 years (58.0%); non-white (76.7%); a large proportion had completed elementary (46.6%) and high school (36.9%); and finally, more than half of the participants received up to the minimum wage (60.8%), as shown in Table 1.

Table 2 presents the perception of the food environment assessed through the application of the NEMS-P. The following aspects that promote healthy eating stand out: fruits and vegetables (FV) available at home and in the neighborhood, good variety and quality of FV in the neighborhood, buying food more frequently, being close to home and places frequented, FV easy to find in the establishment, not buying food near the cashier, not buying food close to eye level, food flavor, nutritional value, weight control, and visiting street markets. On the other hand, some barriers were also perceived: UPF (packages sweets and snacks) available at home, ease of finding UPF (packaged sweets and snacks, sugary drinks) in the establishment, high price of FV, not looking at labels and nutritional information, the most accessible food is unhealthy food, unpalatable FV, poor appearance, promotions in establishments, primarily shopping for food at large supermarket chains, UPF (unhealthy foods) being prominent in establishments, and use of delivery services.

### 3.2. Qualitative

Regarding the qualitative exploration of the data, the sample consisted of 25 participants diagnosed with hypertension, aged between 25 and 89 years old, and most were females (76.0%).

The participants’ reports highlighted several barriers in the perceived food environment that make it difficult to adopt healthier eating habits.

#### 3.2.1. Barriers

##### Disruption of Commensality

Many mentioned the difficulty in maintaining the habit of eating together due to their routines having different times. In addition, they associated the use of technologies such as cell phones and television during meals with a negative eating experience. They reported that eating quickly impairs the taste of the food and social interaction, important factors for valuing healthy eating, since “eating quickly also makes people sick, they spend time looking at the screen, they don’t take advantage of the moment to talk”.

##### Reduction in Home Gardens

The current scarcity of home gardens was highlighted when compared to the past, when it was common to plant for consumption and sale, as observed in statements such as “in the past there was a greater variety of fruits” and “there was a lot of stuff, there was no need to buy”. They reported that fresh foods, such as leafy vegetables, had better flavor and lasted longer. In contrast, fruits and vegetables purchased at grocery stores are seen as less tasty, of lower quality, and more expensive.

##### Economic and Logistical Preference for Shopping in Supermarkets and Recognition of the Impacts of UPF Consumption

Despite the preference for natural foods, participants tend to shop in supermarkets due to lower prices and promotions, even if this requires longer trips and transportation costs, with proximity to home being a secondary factor in choosing where to buy food. Stores close to home showed a high supply of ultra-processed foods (UPF), which were seen as “products that are bad for your health” and which are prominently displayed, negatively influencing food choices. Questions also arose regarding the taste and products used to produce food: “nowadays people, especially young people, are looking for ease, they just want to open packages, cans and plastic bags, the packages are thrown away, which generates waste. All of this generates waste, pollutes the planet, which is our home”.

##### Lack of Time

Lack of time was cited as a limiting factor in both preparing food at home and shopping. Many people do not prepare fresh food, are unfamiliar with the aroma and flavor of home-cooked food, and often resort to delivery for convenience, reducing direct contact with healthy food.

##### Food Transportation and Origin

Reports indicated that a large part of the food sold in the municipality comes from distant supply centers, traveling long distances by road transport, which can influence the quality and freshness of the products.

#### 3.2.2. Promoters

Despite the difficulties, participants also identified factors that favor healthier eating practices.

##### Valuing Street Markets and Local Products

Street markets, with the participation of cooperative farmers from the districts, were recognized as public spaces that promote healthy eating, with fresh, quality products.

##### Positive Domestic Practices—Growing Home Gardens

The importance of changes in the family environment was highlighted, suggesting the organization of meal preparation with pre-preparation of food and encouraging the cultivation of home gardens, taking advantage of available spaces for planting.

##### Concerns About Food Systems

Participants discussed climate change and its impact on local agriculture, highlighting the irregularity of the seasons and the need to reduce the use of pesticides, as well as to think about strategies to minimize food waste.

### 3.3. Intersection of Qualitative and Quantitative Approaches

**Figure 2:**
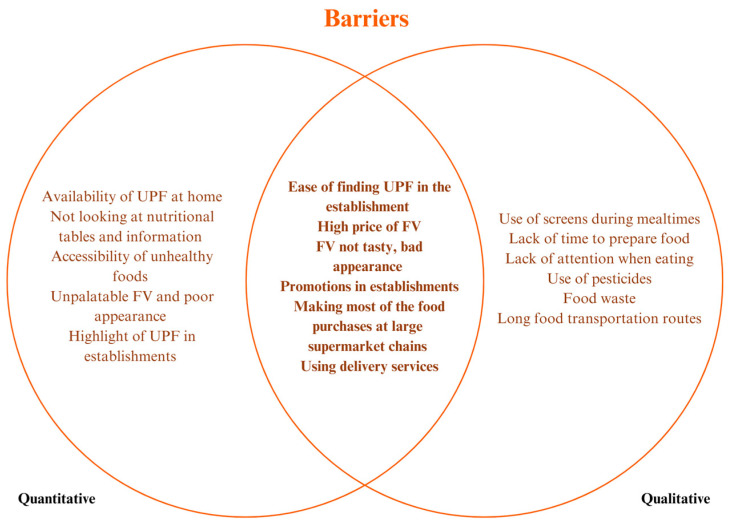
Barriers to adequate nutrition from the food environment perception.

**Figure 3:**
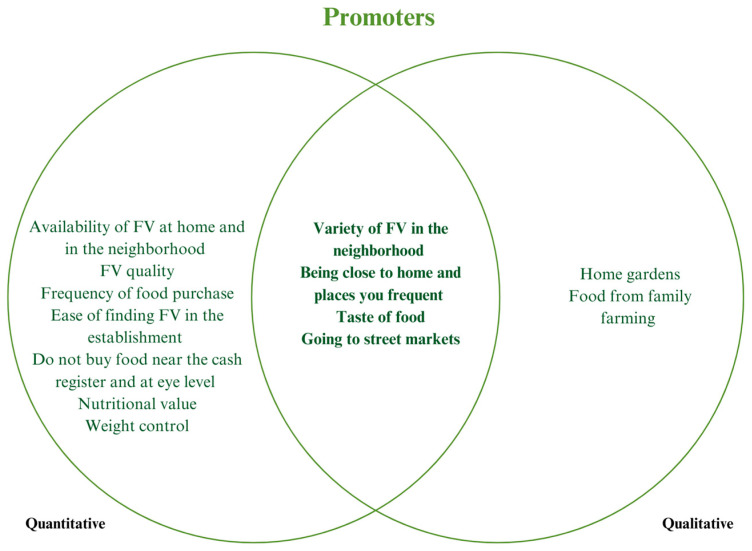
Promoters of adequate nutrition from the food environment perception.

Figure 2 and Figure 3 presents a summary of the barriers and factors that promote healthy eating as perceived by participants, highlighting convergence points between the qualitative and quantitative approaches. The integrated analysis reinforces the importance of understanding the food environment not only through objective indicators but also through people’s experiences, perceptions, and daily practices, thus justifying the adoption of a mixed methodological approach.

Among the most common barriers in both methods, the following stand out: ease of access and visual prominence of UPF in establishments, associated with marketing strategies and shelf placement that encourage consumption, especially near the checkout or at eye level; high price and unsatisfactory appearance of FV, which discourages the purchase of these foods, even among those who recognize their importance for health; frequent promotions of UPF in large supermarket chains, which shape dietary decisions based on price and not on nutritional quality; and increasing use of delivery services, cited as a practical alternative to lack of time, but which reinforces less healthy consumption patterns.

Regarding the promoting aspects, although less mentioned by the participants in the qualitative approach, they partially coincided with the quantitative data, such as variety of FV in the neighborhood, associated with the preference for natural foods, and location close to points of sale, especially street markets, which were perceived as spaces that promote access to fresher, tastier, and locally produced foods and appreciation of the flavor of the food.

**Figure 4:**
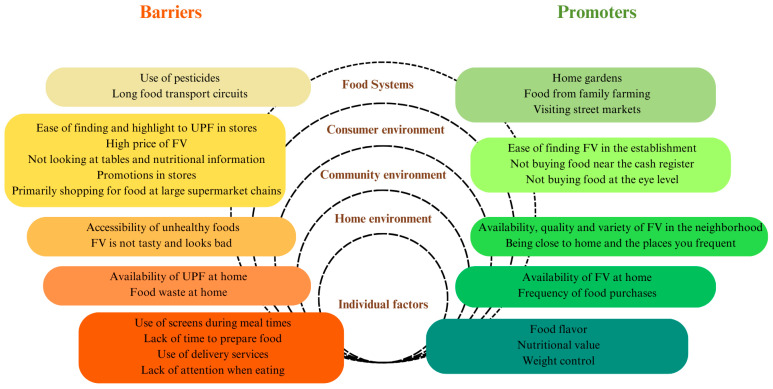
Barriers and promoters of adequate nutrition from the food environment perception.

After analyzing the perceived barriers and promoters, they were grouped without distinguishing between qualitative and quantitative types, as shown in Figure 4. They were then organized according to the dimensions to which they belong, including individual factors, home environment, community environment, consumer environment, and food systems.

## 4. Discussion

This study sheds light on individuals’ perceptions of barriers and promoters of the food environment for healthy eating, with barriers outweighing promoters. Perceptions were similar in both quantitative and qualitative approaches. In general, this study highlighted the high access to UPF and low access to fresh foods, the influence of advertising in establishments, contemporary barriers such as undergoing changes in commensality, and modifications in food systems that lead to worse diets. As for promoters, in short, it was highlighted that access to FV favors better food choices, as well as adds value to the foods, such as flavor and nutrient composition.

### 4.1. Barriers

The main perceived barrier was high access to UPF and low access to fresh foods, both in the community environment (neighborhoods and commercial establishments) and at home. This imbalance can favor the consumption of unhealthy foods [10], as can financial access, which is related to the value attributed to food [27]. The data are corroborated by a systematic review that analyzes the food environment and people in a situation of food insecurity (FI), where households in a situation of FI presented availability of UPF, higher frequency of food purchases in convenience stores and small markets, in addition to lower availability of fresh foods [28]. This scenario is alarming, since the high price of FV and low price of UPF were observed in an analysis of price trends after the COVID-19 pandemic period in Brazil [29], anticipating previous projections [30]. These data reinforce the urgency of public policies aimed at subsidizing healthy foods, such as fruits and vegetables, and regulating prices, especially in contexts of social vulnerability.

In addition to unequal access, another significant barrier was the perception of promotions and advertising focused mainly on UPF at points of sale. Although the information environment plays a crucial role in food choices, current marketing strategies predominantly promote UPF products to the detriment of natural foods [9,31,32]. Although some forms of marketing, such as shelf placement and labels, have not been widely perceived, their impact has already been documented in the literature [33]. However, it is known that label reformulations, such as front-of-pack nutritional labeling, are important tools for information and protecting public health that have proven to be effective. This is demonstrated in modeling studies in Mexico and Brazil, which suggest that the adoption of warning labels can contribute to reducing obesity and containing health costs [34,35]. These findings endorse WHO (2024) recommendations to strengthen regulation of food environments, increasing control over harmful marketing strategies [36].

The changes in the food environment go beyond the dimensions of access and communication, directly reflecting on daily eating practices. The nutritional transition has caused significant changes in commensality, with the progressive replacement of traditional habits—such as home cooking and eating with the family—by eating patterns marked by convenience and consumption outside the home [37,38]. Among the main barriers identified are the use of electronic devices during meals, reduced attention to the act of eating, and the perception that cooking would be a waste of time.

These behaviors negatively impact satiety signals, the quality of chewing, and the emotional bond with the act of eating [39]. In view of this, food and nutrition education programs must incorporate actions that rescue the social, cultural, and emotional value of food, encouraging conscious eating practices and culinary autonomy, in line with the guidelines of the Food Guide for the Brazilian Population [40].

Finally, perceptions about the food environment also reach broader dimensions, such as food systems. In the food system axis, participants reported concerns about the use of pesticides and long marketing channels. Perceptible changes in food—such as taste and appearance—associated with the use of chemicals were mentioned, in addition to possible health impacts. The distance between production and consumption was also perceived as an obstacle to accessing fresh food. These perceptions point to the importance of strengthening short production and marketing channels, such as fairs, community gardens, and institutional purchases from family farming, which promote fresher, more regional, and sustainable food [38]. Encouraging agroecological practices and reconfiguring local food systems should be at the center of strategies to promote health and sustainability.

### 4.2. Promoters

In addition to the barriers identified, this study also revealed important promoters of healthy and sustainable eating, centered around two major axes: qualified access to natural foods and attributes valued in food choices.

The most highlighted positive aspects by participants concern access to FV, both at home and in the community. This positive perception suggests the existence of local infrastructures that favor the consumption of healthy foods, such as fairs, community gardens, and markets offering fresh food. In the production chain, the presence of short marketing circuits was valued, with emphasis on food from family farming, sold at street markets and local initiatives [38]. The importance of these arrangements aligns with the guidelines of sustainable food systems, as they contribute to food sovereignty, the valorization of regional food culture, and the reduction of dependence on agroindustrial chains.

Another driving force refers to the added value of foods that positively influence individuals’ choices. Flavor was widely cited as a preference factor, demonstrating that the pleasure of eating is still a central element in dietary decisions. In addition, nutritional value and potential weight control were mentioned as valued criteria at the time of choice. However, it is important to emphasize that the last two aspects reflect a more nutritionist perspective, which associates foods with their physiological properties, sometimes disregarding their symbolic, cultural, and social meanings [41]. This perception may be a reflection of the influence of biomedical discourses on food and health. Thus, there is an opportunity for PHC to act in order to expand the repertoire of healthy eating, promoting a vision that integrates pleasure, culture, tradition, and health in a more holistic way.

It is worth noting that participants perceived different dimensions of the food environment, which go beyond physical and economic access. They identified issues ranging from individual issues, such as contemporary commensality marked by isolation and convenience, to more symbolic and structural elements, such as nutritionism—where food is seen only for its physiological function—and aspects linked to the food system, among which the use of pesticides and the recognition of more sustainable production chains, such as family farming.

This study has some limitations that should be considered. First, the use of different samples for the quantitative stage may have influenced the results, limiting direct comparability between the groups analyzed. It is also worth noting that the small number of participants in the focus groups, although appropriate for the qualitative approach and the theoretical saturation criterion, may not represent the full complexity of the population under study. Furthermore, the factors that determine participants’ perceptions of the environment and the food system, both in positive and negative terms, were not investigated. Another relevant point is that cultural diversity among participants, especially with regard to urban or rural origin, was not specifically explored, which may have impacted the perceptions reported. The absence of these analyses prevents a more in-depth understanding of the reasons underlying the perceptions identified, which could enrich the implications of this study for intervention strategies.

Despite its limitations, this study analyzes individuals’ perceptions, therefore assessing the food environment from a subjective perspective, standing out among the various studies that assess the environment using objective methodologies. It also advances when it integrates qualitative and quantitative methodologies of subjective methods, highlighting that in both, it is possible to note the similarity of the responses. Another relevant point is the articulation between the environment and the food system, expanding the understanding of the determinants of healthy eating in specific territorial contexts. And another important limitation of the present study concerns the adaptation of the instrument used to assess perceptions of the food environment. Modifications were made to the original scale, including the removal of some items, without conducting psychometric analyses of the revised version. The absence of validity and reliability indicators, such as Cronbach’s alpha, limits the methodological robustness of the findings. Future studies are recommended to validate the adapted scale in order to ensure its internal consistency and contextual appropriateness. A limitation of this study is that the classification of items from the quantitative component as barriers or facilitators was based on theoretical interpretation, rather than being directly reported by the participants.

Future studies should use samples that are more integrated between qualitative and quantitative approaches in order to allow for more consistent comparisons between data. Future research should also explore the factors that determine perceptions about the environment and the food system in depth, including cultural, social, and territorial elements, such as the distinction between urban and rural contexts. Such analyses can contribute to a broader and more contextualized understanding of the perceived barriers and promoters of healthy and sustainable eating. Considering that this study identified a predominance of barriers—such as high access to ultra-processed foods, the influence of advertising, and changes in eating patterns—to the detriment of promoters, it is essential to further investigate the determinants of these perceptions in order to support more effective and culturally sensitive policies and interventions.

## 5. Conclusions

Understanding how individuals perceive the environment in which they live and make food choices is of utmost importance, going beyond objective assessment. This study observed that individuals perceive high access to UPF, both through financial means and availability, as well as low access to fresh foods. These findings reinforce the importance of intersectoral policies that simultaneously address health, food security, sustainability, and social justice. Integrating the data reveals that to promote healthier and more sustainable food environments, it is necessary not only to guarantee physical and economic access to healthy foods but also to create conditions that favor more conscious, participatory, and territorially rooted eating practices, as well as policies that encourage the commercialization of fresh foods and the taxation of UPF, affecting the final price.

## Figures and Tables

**Table 1:** Socioeconomic characteristics of patients diagnosed with hypertension in the city of Ouro Preto, MG.

Variables	Total (n = 176)	100%
**Sex**		
Female	139	79.0
Male	37	21.0
**Age range**		
20 to 39 years	74	42.0
40 to 59 years	102	58.0
**Skin color**		
White	41	23.3
Non-white	135	76.7
**Education**		
Elementary	82	46.6
Secondary	65	36.9
**Salary range**		
1–2 minimum wages	107	60.8
>2 minimum wages	69	39.2

**Table 2:** Characteristics of the perceived food environment of patients diagnosed with arterial hypertension.

Variables	Answer Options	Total (n = 176)	100%
**Home food environment**			
There are FV * in the fridge	Frequently	144	82.8
	Rarely	32	18.2
There are packaged sweets and snacks	Frequently	27	15.3
	Rarely	149	84.7
**Community food environment**			
It’s easy to buy FV in the neighborhood	I agree	137	77.8
	I disagree	39	22.2
It’s easy to find good quality FV in the neighborhood	I agree	142	80.7
	I disagree	34	19.3
It’s easy to find many options of FV in the neighborhood	I agree	129	73.3
	I disagree	47	26.7
Frequency of food purchases	1x/week, >1x/week, 1x/2 weeks	124	70.4
	Once a month	42	23.9
	Other	10	5.7
Type of establishment where you buy your food	Supermarket/hypermarket	150	85.2
	Grocery store, warehouse or market	20	11.4
	Fair or greengrocer’s market	6	3.4
Time spent walking to the establishment where you buy most food	10–20 min	72	41
	>21 min	104	59
Establishment is close to your home	Important	126	71.6
	Not important	50	28.4
It is close to or on the way to other places you frequent	Important	122	69.4
	Not important	52	19.5
Your friends/family shop at this establishment	Important	81	49.5
	Not important	94	50.5
Variety of food options at this establishment	Important	166	94.4
	Not important	10	5.6
Quality of food at this establishment	Important	173	98.3
	Not important	3	1.7
Price of food at this establishment	Important	164	93.2
	Not important	12	6.8
**Consumer food environment**			
Ease of finding FV at the establishment	Easy	138	78.4
	Hard	38	21.6
Ease of finding packaged sweets and snacks in the store	Easy	170	96.6
	Hard	6	3.4
Ease of finding sugary drinks in the store	Easy	171	97.2
	Hard	5	2.8
Price assessment of FV where most of the food is purchased	Cheap	63	35.8
	Expensive	70	39.8
	No fruits and vegetables available	27	15.3
	Fair price for good quality	8	4.5
	Unfair price for poor quality	8	4.5
I notice signs encouraging me to buy healthy foods	I agree	51	29
	I disagree	125	71
I often buy food near the checkout counter	I agree	20	12.4
	I disagree	154	87.6
Unhealthy foods are usually near the end of the aisles	I agree	58	33.3
	I disagree	116	66.7
I often buy items that are at eye level on the shelves	I agree	47	26.9
	I disagree	128	73.1
There are many signs or displays encouraging me to buy unhealthy foods	I agree	77	44
	I disagree	98	56
I look at the nutritional information tables on most food packages in stores	I agree	59	33.7
	I disagree	116	66.3
I do my shopping through delivery apps and I feel influenced to buy unhealthy foods	I agree	20	11.4
	I disagree	155	88.6
**Habits and thoughts about food**			
When buying food, taste matters	Important	173	98.3
	Not very important	3	1.7
When buying food, the nutritional value	Important	153	86.9
	Not very important	23	13.1
When buying food, cost matters	Important	172	97.7
	Not very important	4	2.3
When buying food, the ease of finding the food matters	Important	165	94.3
	Not very important	10	5.7
When buying food, the weight control matters	Important	164	93.2
	Not very important	12	6.8

* FV: fruits and vegetables.

## Data Availability

All data are available in the article.

## Abbreviations

The following abbreviations are used in this manuscript:

AH	Arterial Hypertension
BHU	Basic Health Units
FI	Food Insecurity
FV	Fruits and Vegetables
FHS	Family Health Strategies
NCDs	Chronic Non-Communicable Diseases
NEMS-P	Perceived Nutrition Environment Measures Survey
PHC	Primary Health Care
UPF	Ultra-Processed Foods
